# Growth and carcass characteristics of three Ethiopian indigenous goats fed concentrate at different supplementation levels

**DOI:** 10.1186/s40064-016-2055-2

**Published:** 2016-04-06

**Authors:** Dereje Tadesse, Mengistu Urge, Getachew Animut, Yoseph Mekasha

**Affiliations:** Debre Berhan University, P.O. Box 445, Debre Berhan, Ethiopia; Haramaya University, P.O. Box 138, Dire Dawa, Ethiopia; International Livestock Research Institute, P.O. Box 5689, Addis Ababa, Ethiopia

**Keywords:** Dietary levels, Goat breed, Growth rate, Carcass cuts, Linear measurement

## Abstract

The study was carried out to evaluate the effect of genotypes and concentrate levels on growth performance and carcass characteristics of Bati, Hararghe highland (HH) and Short eared Somali (SS) goat types found in Ethiopia. A 3 × 2 factorial arrangement (3 genotype × 2 concentrate levels) was used to randomly allocate 36 goats (15.2 ± 0.30 kg initial weight); 12 goats from each genotype with age about 1 year were divided randomly into two groups for a feeding trial of 90 days. The two concentrate levels were L1 and L2, where L1 and L2 are levels fed to animals at the rate of 1 and 1.5 % BW, respectively. Hay was fed ad libitum with 20 % refusal rate. The mean daily dry matter intake of the goats was 520.5 g/day. The intake was about 67 g/day higher for L2 than L1 goats. Consequently, L2 goats had significantly (p < 0.05) higher average daily gain, dressing percentage, primal carcass cuts and total non-carcass fat than those fed L1. Among genotypes, HH goats were found to have higher (p < 0.05) carcass weight, heart girth, neck girth, and carcass cuts (legs and shoulders) than SS goats. However, they were not better in dressing percentage than SS goats. Compared to Bati goats, HH goats had significantly (p < 0.05) wider rib-eye area, heavier ribs/racks weights, and better dressing percentage. Despite smaller body size, the performance of SS goats was comparable to Bati goats. In conclusion, the study indicates the potential of Ethiopian indigenous goats to produce optimum amount of meat when supplemented with concentrate at the rate of 1.5 % body weight.

## Background

Small ruminants play a significant role in almost all farming systems in the tropics and sub-tropics (Anaeto et al. 2009). A recent report shows that Ethiopia has the largest goat population (24.1 million) among African countries (CSA 2013). Goats are primarily kept for meat production, although milk is equally important in pastoral and agro-pastoral areas. They are usually raised and finished on natural pastures, and as a result they take a long time (over 2 years) to reach slaughter weight not more than 20 kg. The carcass weight produced is not higher than 8.5 kg per goat (FAO 2004).

Many reports indicate that goats fed with low quality roughage have satisfactory fattening performance when supplemented with concentrate having optimum contents of CP and energy (Mushi et al. 2009a). It reduces age to slaughter, increases carcass quality and meat output thereby improving access to animal protein and income to households in the subsistence production system (Mtenga and Kitaly 1990).

A number of research works (Webb et al. 2005; Wondwosen et al. 2010; Goetsch et al. 2011; Wallie et al. 2012; Tilahun et al. 2013; Tadesse et al. 2013) have been conducted to assess the effect of plane of nutrition on growth and carcass performance of a particular goat genotype. However, there are limited information on whether the growth and carcass characteristics of Ethiopian indigenous goats are differently influenced by genotype and nutritional regimes (Ameha et al. (2007). They have not been compared and characterized adequately in terms of their growth, carcass and meat quality attributes.

In order to effectively use their potential, it is vital to understand their growth performance and carcass characteristics, their response to different feeding regimes and their possible integrations into various production systems. The objective of this study was to evaluate the effects of genotype and levels of concentrate on growth performance, carcass characteristics and non-carcass yield of Ethiopian indigenous goats.

## Methods

### The study area

The study was conducted at Haramaya University Goat Farm in Ethiopia, which is located at 42°E longitude and 9°N latitude. The area is located 515 km east of Addis Ababa and found at an altitude of 1950 m above sea level. It has an average temperature of 16 °C with mean annual temperature ranging between 9.73 and 24.02 °C. The area has a bimodal type of rainfall and receives an average annual total rainfall of 790 mm (Mishra et al. 2004).

### Animal management and experimental design

Though Ethiopia had no established system for ethical approval of animal experiments, all procedures involving the animals followed the international guiding principles listed by the Council for International Organizations of Medical Sciences and the International Council for Laboratory Animal Science (2012).

Thirty-six yearling Ethiopian indigenous goats (15.2 ± 0.30 kg) belonging to three genotypes were used in this study. The three genotypes used were Bati, Hararghe highland (HH) and Short-eared Somali (SS) goats. They have been well described in the work of FARM-Africa (1996). The goats were purchased from local markets and transported to Haramaya University. They were quarantined for 3 weeks being vaccinated for diseases such as ovine pasteurellosis, sheep and goat pox and anthrax. They were also injected with Ivermectin, dewormed with Albendazole, and sprayed with acaricide against parasites. After quarantine, each animal was kept in an individual pen with 1.2 × 0.7 m dimension and acclimatized for 2 weeks.

A 3 × 2 factorial arrangement (3 genotypes × 2 concentrate levels) was used to randomly allocate 36 goats. Twelve goats from each genotype were divided randomly into two groups for a feeding period of 90 days. The two concentrate levels were L1 and L2, where L1 and L2 are levels fed at the rate of 1 and 1.5 % of body weight, respectively. Natural pasture hay dominated with grass species such as *Digitaria nodosa*, *Sporobolus natalensis* and *Eragrostis papposa* was fed ad libitum at 20 % refusal rate. The concentrate comprised of wheat bran (57 %), noug cake (23 %), maize grain (19 %) and salt (1 %) on DM basis. It was formulated to contain CP and energy to meet the optimum recommendation of Flint (2005) for intensive feeding (i.e. 18 % CP and 9 MJ ME/kg DM). Table 1 shows the actual laboratory results of chemical compositions of the feeds used in this study. The concentrate was given in two equal meals at 08:00 and 14:00 h and the amount was adjusted based on body weight every 10 days. Clean water was available all the time.

**Table 1:** Chemical composition (%) of experimental feeds used in the study

Experimental feeds	DM	OM	Ash	CP	NDF	ADF
Hay	90.5	88.8	11.2	8.1	80.3	48.7
Concentrate	89.2	93.7	6.3	21.0	37.9	15.7
Wheat bran	89.9	94.9	5.1	17.9	47.4	14.4
Noug cake	91.6	91.7	8.2	38.6	30.5	22.9
Maize	88.1	98.2	1.8	7.6	25.2	7.9

*DM* dry matter, *OM* organic matter, *CP* crude protein, *NDF* neutral detergent fibre, *ADF* acid detergent fibre

### DM and nutrient digestibility

At the end the feeding trial, all animals were made to adapt carrying the fecal bags for 3 days followed by total collection of feces for 7 days. The feces collected every day from each animal were weighed, thoroughly mixed, 10 % sampled and stored in a refrigerator at −20 °C. The feces sampled for 7 days were pooled for each animal, thoroughly mixed and then 10 % sub-sampled for chemical analysis.

### Feed and growth measurements

The weight of concentrate and hay offered and refused were recorded daily to derive feed intake. Animals were weighed every 10 days before morning feeding. Initial weight (IW) and final weight (FW) were recorded twice for two consecutive days and the average was taken. Average daily gain (ADG) was determined by regressing body weight against time. Feed conversion ratio (FCR) was calculated for each animal as proportion of DM intake to weight gain (kg DM intake per kg weight gain).

Body linear measurements such as body length (length from point of shoulder to pin bone), height at withers (measured from base of hoof to highest point of wither), heart girth (body circumference immediately behind the forelegs), neck girth (circumference at base of neck), pelvis width (distance between the two hocks) and thigh circumference (circumference around the middle of the thigh) were taken at the end of the feeding trial using measuring tape as described by De Boer et al. (1974).

### Slaughter procedure and carcass measurements

At the end of the study, all goats were fasted for 12 h overnight with free access to water. Then the goats were slaughtered at Haramaya University slaughterhouse after taking their slaughter body weight (SBW). Hot carcass weights (HCW) were recorded immediately after slaughter. Edible (heart, liver, kidney, blood, digestive organs, total fat) and nonedible (skin, head, feet, spleen, lungs, trachea, genitalia) carcass components were weighed and recorded. The digestive tract was weighed when full and empty in order to compute the weight of intestinal content/digesta. Empty body weight (EBW) was computed as the difference between SBW and the weight of the digesta while dressing percentage (DP) was calculated as HCW/SBW * 100 and HCW/EBW * 100.

Carcass linear measurements such as carcass length (measured from caudal edge of the last vertebrae to the dorso-cranial edge of the atlas), leg width (widest measurement of hind leg), chest depth (maximum distance between the sternum and the back of the carcass at the sixth thoracic vertebra) and shoulder width (maximum width of the shoulder measured at the level of the scapula from one lateral surface to the other) were taken following the method of De Boer et al. (1974). Eye-muscle area and fat thickness were measured at the 12/13th rib position. The left and right rib-eye muscle area was traced onto a paper and the area of the squares that fell within the traced area was counted and those partially fell outside was estimated. The average of the two sides was taken as the rib-eye muscle area. The carcass was partitioned into four primal cuts (legs, ribs/rakes, lion, neck and shoulder) to determine their proportion as described by Smith et al. (1978). The weight of total internal fats (fats from the kidney, scrotal, pelvic, and heart) was recorded using sensitive balance.

### Chemical analysis of feeds

Samples of feeds and feces were dried at 65 °C for 48 h. The dried samples were then ground (1 mm screen) and stored for subsequent analyses of dry matter (DM), crude protein (CP), ash, neutral detergent fibre (NDF) and acid detergent fibre (ADF). DM, N and total ash were determined according to the official methods of AOAC (1990) and NDF and ADF according to Van Soest et al. (1991). Dry matter content of the feed was determined by drying the samples in an oven at 105 °C overnight while ash content was determined by burning the samples at 550 °C for 5 h in a muffle furnace. Nitrogen (N) was determined by Kjeldahl method (CP = N × 6.25).

### Statistical analysis

The data were analyzed using general linear model (PROC GLM) procedure of SAS (2003) to determine the influence of main effects on all dependent variables considered. The statistical model used was: Y_ijkl_ = µ + B_i_ + G_j_ + F_k_ + (G × F)_jk_ + E*ijkl.* Where; Y_ijkl_ = the response variable; μ = overall mean; B_i_ = effect of block; G_j_ = effect of genotype; F_k_ = effect of feeding level; (G × F)_jk_ = interaction between genotype and concentrate level, and E_ijkl_ = random error. Least squares means were estimated, and mean separation was performed using adjusted Tukey Test when F test was found to be significant (p < 0.05). Since interactions were not significant for all parameters, means for main factors were presented and discussed.

## Results

### Intake and digestibility

Least square means for intake and apparent digestibility of DM and nutrients, and feed conversion ratio (FCR) of goats are presented in Table 2. Daily dry matter intake from concentrate was higher for goat receiving L2 than L1. Similar trend was observed for total daily dry matter. Goats receiving L2 supplement consumed 67 g higher (p < 0.01) total DM than that of L1 goats. Differences in hay DM intake among genotype and between concentrate levels were not apparent. Daily concentrate DMI of Bati was significantly higher than SS goats. However, total DM intake expressed as percentage of body weight was higher (p < 0.05) for SS goats than Bati goats. The CP intake (%BW) and digestibility of DM, OM and CP were also significantly higher for L2 goats. About 11.7 and 13.5 kg of feed were consumed by L2 and L1 goats per one kg of weight gain, respectively during the experimental period. However, difference in FCR among genotypes was not significant (p > 0.05) though HH and SS goats tended to have better efficiency than Bati goat.

**Table 2:** Least square means for feed intakes, digestibility, and conversion efficiency of three Ethiopian goat types

Variables	Genotype (G)			Concentrate level (C)			*p* value		
	Bati	HH	SS	L1	L2	SEM	G	C	G × C
*DM intake*									
Concentrate (g/day)	200.4^a^	196.0^ab^	179.3^b^	150.3^b^	233.5^a^	8.28	0.02	<0.01	0.88
Hay (g/day)	323.5	344.9	317.5	336.7	320.5	6.20	0.12	0.15	0.78
Total (g/day)	523.8	540.9	496.8	487.1^b^	553.9^a^	11.2	0.09	<0.01	0.92
Total (%BW)	2.86^b^	2.96^ab^	3.01^a^	2.82^b^	3.06^a^	0.03	0.03	<0.01	0.18
*CP intake*									
Concentrate (g/day)	42.1^a^	41.1^ab^	37.6^b^	31.5^b^	48.9^a^	1.74	0.02	<0.01	0.88
Hay (g/day)	26.1	27.7	25.6	27.2	25.8	0.49	0.12	0.15	0.78
Total (g/day)	68.1	68.9	63.2	58.5^b^	74.8^a^	1.89	0.05	<0.01	0.96
Total (%BW)	0.37	0.37	0.38	0.34^b^	0.41^a^	0.01	0.16	<0.01	0.05
*Digestibility (%)*									
Dry matter	71.7	68.3	69.1	67.3^b^	72.1^a^	1.03	0.29	0.01	0.29
Organic matter	72.3	68.9	69.7	67.9^b^	72.7^a^	1.02	0.28	0.01	0.29
Crude protein	77.4	74.3	74.6	73.2^b^	77.6^a^	0.87	0.18	<0.01	0.23
NDF	67.5	63.8	64.2	63.5	66.8	1.16	0.33	0.14	0.45
ADF	54.9	50.5	51.8	51.4	53.4	1.44	0.43	0.47	0.39
FCR	14.2	10.8	12.8	13.5	11.7	0.62	0.09	0.17	0.79

Least square means in a row with different subscript letter differ significantly (p < 0.05)

*HH* Hararghe highland goat, *SS* Short-eared Somali goat, *L1* hay + 1 % of body weight concentrate, *L2* hay + 1.5 % of body weight concentrate, *FCR* feed conversion ratio, *SEM* pooled standard error of mean

### Body growth and dimensions

Average daily gain (ADG) was significantly (p < 0.05) affected by level of concentrate but not by genotype (Table 3). Goats fed L2 gained 1 kg extra body weight compared to those fed L1 by growing at an average of 12 g greater rate per day. With increasing DM intake, there was a positive linear increase (r = 0.63; p < 0.01) in body weight change of animals. Bati and HH goats were heavier (p < 0.05) by more than 2 kg in their final weight (FW) and slaughter body weight (SBW) than SS goats. They also had higher (p < 0.05) height at withers and neck girths compared to SS goats. For other body linear measurements such as body length, heart girth and pelvic width, no significant differences were evidenced among genotype. Goats fed L2 had wider (p < 0.05) pelvic width than those consumed L1. They also tended (p = 0.10) to have better height at wither than L1 goats.

**Table 3:** Least square means for body weight and dimensions of three Ethiopian goat types

Variables	Genotypes (G)			Concentrate level (C)		SEM	p value		
	Bati	HH	SS	L1	L2		G	C	G × C
*Growth parameters*									
IW (kg)	15.8	15.5	14.1	14.9	15.3	0.30	0.11	0.29	0.12
FW (kg)	19.6^a^	20.1^a^	17.8^b^	18.4^b^	19.9^a^	0.38	<0.01	0.02	0.48
ADG (g/day)	42.1	51.4	41.3	38.7^b^	50.9^a^	2.42	0.17	0.02	0.98
SBW (kg)	18.8^a^	19.3^a^	17.1^b^	17.8^b^	19.0^a^	0.37	<0.01	0.02	0.53
*Dimensions (cm)*									
Body length	51.7	51.2	50.5	50.7	51.6	0.38	0.47	0.32	0.96
Heart girth	65.8	65.9	64.3	64.9	65.8	0.41	0.19	0.28	0.84
Height at wither	60.8^a^	61.4^a^	58.4^b^	59.6	60.8	0.46	<0.01	0.10	0.71
Pelvic width	10.7	11.0	10.5	10.4^b^	11.1^a^	0.16	0.34	0.04	0.61
Neck girth	34.0^a^	34.0^a^	32.7^b^	33.4	33.7	0.24	0.03	0.51	0.53
Thigh circumference	20.5	20.8	20.3	20.3	20.8	0.15	0.34	0.12	0.53

Least square means in a row with different subscript letter differ significantly (p < 0.05)

*HH* Hararghe highland goat, *SS* Short-eared Somali goat, *L1* hay + 1 % of body weight concentrate, *L2* hay + 1.5 % of body weight concentrate, *IW* initial weight, *FW* final weight, *ADG* average daily gain, *SBW* slaughter body weight, *SEM* pooled standard error of mean

### Carcass weight and dimensions

Least square means for carcass weight and linear measurements of goats kept under two levels of feeding are set in Table 4. HH goats had higher (p < 0.05) EBW and HCW than SS goats but had comparable carcass weight with that of Bati goats. The dressing percentage (DP) and rid-eye area of HH goat was higher DP of Bati goats but not different from that of SS goats. The DP of L2 goats was about 2.5 % higher than that of L1 goats. The average proportion of the leg relative to the weight of the carcass was about 32.6 % and varied between supplemented groups. A comparison between Bati and SS goats indicated that SS goat had significantly lighter fore-quarter part or shoulder. Goats receiving L2 had higher (p < 0.01) proportion of legs and ribs/racks compared to those receiving L1.

**Table 4:** Least square means for slaughter and carcass characteristics of three Ethiopian goat types

Variables	Genotype (G)			Concentrate level (C)		SEM	p value		
	Bati	HH	SS	L1	L2		G	C	G × C
*Carcass wt*									
EBW (kg)	15.2^a^	15.6^a^	13.6^b^	14.2^b^	15.5^a^	0.36	<0.01	<0.01	0.69
HCW (kg)	7.8^ab^	8.3^a^	7.2^b^	7.3^b^	8.4^a^	0.22	<0.01	<0.01	0.21
DP (EBW basis)	51.2^b^	54.4^a^	53.0^ab^	51.8^b^	53.9^a^	0.61	0.04	0.03	0.07
DP (SBW basis)	41.5^b^	43.9^a^	41.9^ab^	41.1^b^	43.8^a^	0.54	0.03	<0.01	0.16
*Dimensions (cm)*									
Carcass length	50.9	47.8	45.4	47.7	48.4	1.01	0.10	0.74	0.74
Chest depth	28.4	28.2	27.1	27.4	28.3	0.30	0.12	0.11	0.44
Shoulder width	16.7	16.8	15.5	15.8	16.9	0.80	0.77	0.49	0.18
Hind leg width	11.1^ab^	11.9^a^	10.7^b^	11.2	11.3	0.19	0.02	0.75	0.84
Rib-eye area (cm^2^)	6.9^b^	9.8^a^	8.8^ab^	7.9	9.1	0.31	<0.01	0.06	0.43
Fat thickness	1.14	1.17	1.22	1.1	1.2	0.03	0.45	0.09	0.77
*Primal cuts (%)*									
Legs	32.5	32.1	33.2	32.1^b^	33.2^a^	0.35	0.41	0.91	0.35
Loin	10.3	10.5	10.4	10.2	10.5	0.32	0.99	0.94	0.61
Ribs/racks	11.6^b^	13.9^a^	13.8^a^	12.6^b^	13.6^a^	0.37	0.01	0.04	0.36
Shoulder	45.5^a^	43.5^ab^	42.5^b^	43.3	44.7	0.44	0.02	0.24	0.43

Least square means in a row with different subscript letter differ significantly (p < 0.05)

*HH* Hararghe highland goat, *SS* Short-eared Somali goat, *L1* hay + 1 % of body weight concentrate, *L2* hay + 1.5 % of body weight concentrate, *SBW* slaughter body weight, *EBW* empty body weight, *HCW* hot carcass weight, *DP* dressing percentage, *SEM* pooled standard error of mean

### Non-carcass components

Table 5 shows least square means for non-carcass components of three Ethiopian goat types. The effects of genotype and feeding level were significant (p < 0.05) for some of the traits such as liver, heart plus lung, head, skin plus feet and total non-carcass/offals. SS goats had lower (p < 0.05) liver, skin plus feet, total offals and edible offals than Bati and HH goats. Goats fed L2 had heavier (p < 0.01) liver, heart plus lung, skin plus feet and empty GIT than those fed L1. In terms of fat deposition, SS goat which represented the lowland pastoral/agro-pastoral goat types had about 40 g heavier total internal fat than Bati and HH goats. Total non-carcass fat depositions obtained from goats receiving L2 were nearly twofold higher compared to those from goats fed L1. Bati and HH goats had heavier skins and feet than SS goats. The difference was also reflected between feeding levels where animals given L2 had significantly heavier skins and feet, total offals and edible offals than those receiving L1.

**Table 5:** Least square means for non-carcass components (kg) of three Ethiopian goat types

Non-carcass traits	Genotype (G)			Concentrate level (C)		SEM	p value		
	Bati	HH	SS	L1	L2		G	C	G × C
Kidney	0.1	0.06	0.05	0.05	0.1	0.001	0.06	0.16	0.73
Liver	0.3^a^	0.3^a^	0.25^b^	0.26^b^	0.34^a^	0.01	<0.01	<0.01	0.67
Heart + lung	1.0^a^	0.9^ab^	0.9^b^	0.89^b^	0.99^a^	0.03	0.02	0.04	0.14
Head	1.2	1.3	1.09	1.2	1.21	0.03	0.05	0.51	0.62
Spleen	0.03	0.02	0.03	0.03	0.03	0.002	0.28	0.49	0.30
Skin + feet	2.5^a^	2.5^a^	2.2^b^	2.3^b^	2.5^a^	0.05	<0.01	<0.01	0.20
GIT empty	1.2	1.3	1.2	1.1^b^	1.28^a^	0.03	0.25	<0.01	0.89
Digestive content	3.5	3.7	3.4	3.5	3.53	0.11	0.61	0.82	0.28
Genitalia	0.24	0.22	0.23	0.22	0.24	0.01	0.78	0.06	0.13
Non-carcass fat	0.29	0.31	0.35	0.23^b^	0.40^a^	0.02	0.25	<0.01	0.69
Total offals	10.4^a^	10.8^a^	9.7^b^	9.9^b^	10.6^a^	0.19	0.02	0.03	0.40
Total edible offals	3.01^a^	3.02^a^	2.72^b^	2.7^b^	3.1^a^	0.06	0.02	<0.01	0.83

Least square means in a row with different subscript letter differ significantly (p < 0.05)

*HH* Hararghe highland goat, *SS* Short-eared Somali goat; *L1* hay + 1 % of body weight concentrate, *L2* hay + 1.5 % of body weight concentrate, *GIT* gastro-intestinal tract, *SEM* pooled standard error of mean

## Discussion

### Feed and nutrient intakes

The daily DM intake (% live weight) of goat in this experiment is within the range of 2.6–3.2 % reported for Small East African goat and their crosses with Norwegian goat fed hay and supplemented with concentrate (William et al. 2013). For indigenous Ethiopian goats, DM intakes in the ranges of 2.9–3.9 and 2.9–3.2 % body weight were reported for Sidama (Solomon et al. 2008; Wondwosen et al. 2010; Tadesse et al. 2013) and for Hararghe highland goats (Wallie et al. 2012), respectively when they were supplemented with different levels of concentrates and other materials such as sweet potato vines and khat leftovers. Different from previous works, however, hay DM intake of goat in the present study was not significantly dropped as level of concentrate increased. This could be attributed to the relatively better quality of the hay which contained CP higher than the minimum of 70 g/kg DM CP required to support optimal microbial activity in the rumen (McDonald et al. 2002). Van Soest (1994) noted a rapidly declining trend of feed intake when the CP content of the consumed roughage is falling below 70 g/kg DM of the feed.

The reason that L2 animals consumed more total DM and had better feed conversion efficiency is because they ingested about 27.4 % more CP and had higher rate of DM, OM and CP digestibility than L1 animals. As reported by Goodchild and McMeniman (1994), supplementation of poor quality forage with good protein feed has increased the availability of nitrogen in the rumen, thereby improving the rate of degradation and utilization of the feed. The average total CP intake of goats in the present study is in line with the recommended requirements of 65–70 g/day CP for goats weighing 20–25 kg (Kearl 1982). This indicates that the ration used in the present study can support better performance in goats. The estimated daily energy intakes were in the range of 4.8–5.9 MJ ME, which are within the recommended range of 3.25–6.47 MJ (Devendra and Burns 1983).

### Growth performance

The difference in growth performance of goats fed different concentrate levels in the present study reflects the variations in feed conversion efficiency. The extra 1 kg weight gain of L2 over L1 goats can be due to their increased DM and protein intakes. The average daily weight gain recorded in the present study is within the range of 29–51 g reported for Small East African goats fed low and high levels of concentrate (Hango et al. 2007). Daily weight gains of 33–49 g/day were reported for HH goats fed different levels of leftover khat (*Catha edulis*) (Wallie et al. 2012), which are very close to the present values. Higher growth rates were also reported for Somali (Mengistu et al. 2007; Solomon and Simret 2008) and Sidama goats (Solomon et al. 2008) supplemented with concentrate and fed hay as basal diet.

Body linear dimensions such as heart girth, height at wither, and body lengths are reported to be closely related to the size or weight of an animal (Vargas et al. 2007). In present study, only height at wither was found to be significantly higher for Bati and HH goats than SS goats, probably due to the fact that these goats are naturally taller than SS goats (FARM-Africa 1996). However, supplementation of goats with low to moderate quality basal diets did not affect body length and heart girth. The fact that SS goats had shorter height (p < 0.05) but comparable body length and heart girth with that of Bati goats indicates that SS goats have compact body than Bati goats. This is also confirmed by the result that SS goats tended to have relatively higher DP than Bati goats (Table 4). Mahgoub and Lu (1998) reported that Dhofari goat with smaller body size had higher carcass muscularity than Batina goat of larger body size.

### Carcass characteristics

The carcass weight recorded in the present study is within the carcass weight of 6–9 kg reported for other Ethiopian goats (Hailu et al. 2005; Ameha et al. 2007). The effect of diet on carcass weight was clearly seen. The difference in carcass weight between supplement groups may be attributed to higher feed intake and, consequently, greater nutrient availability to promote weight gain and tissue development in goats fed L2 diet. Mushi et al. (2009a) also reported higher weights and carcass yields in goats fed higher energy levels.

The dressing percentage, expressed as percentage of full and empty body weight, were 42.4 and 52.8 %, respectively. The values are within the range of 39.5–41.8 and 53.3–56.6 % reported for Ethiopian (Ameha et al. 2007) and Omani goat breeds (Kadim et al. 2003). The reason for variation in DP among genotypes in the present study is because of greater difference in proportion of non-carcass components such as GIT. Dhanda et al. (1999) and Addisu et al. (2002) also reported a significant effect of genotype on DP and attributed this difference to variations in weight of digestive tract contents. On the other hand, the higher DP of L2 goat compared to that of L1 goat is likely associated to better development of muscle and fat tissue due to their increased DM and CP intakes (Safari et al. 2011).

According to De Boer et al. (1974) linear carcass measurements are indices indirectly help to determine carcass conformation and affected by genotype, sex and feeding regimes. HH and SS goats had relatively wider (p < 0.05) rib-eye area than Bati goats implying that they have more desirable carcass. According to the report of Wolf et al. (1980), larger rib-eye area is associated with higher production of lean in the carcass and higher lean to bone ratio. The proportion of the legs (32–33 %) calculated in the present study is within 28–33 % reported for well-conformed goat breeds (Sen et al. 2004; Webb et al. 2005). The reason for SS goats to have equal proportions of primal cuts compared to that of Bati goat is because they deposited more muscle tissue relative to their body size. Mahgoub and Lu (1998) noted that small body sized goats had a higher carcass muscularity than large body sized ones.

### Non-carcass characteristics

Information on yield of non-carcass components of animals is important especially in developing countries where they are more valuable for the households (Mushi et al. 2009b). The presence of variations in internal fat deposition among different breeds of goat has been reported earlier by Mahgoub and Lu (1998) and Kadim et al. (2003). On average, about 0.32 kg total non-carcass fat was recorded in the present study, which is lower than 0.57 kg reported for other Ethiopian indigenous goats (Ameha et al. 2007). Among the three goat types, SS goats can be regarded as physiologically early maturing animals as they have higher deposition of internal fats, and also better composition of chemical fat in the muscle as indicated by Tadesse et al. (2015). According to Ameha et al. (2007), different animals can have different physiological maturities as a result of difference in chemical fat composition and total non-carcass fat contents.

Higher proportions of total and edible offals were obtained from L2 than L1 goats, which is in agreement with the results of Solomon et al. (2008) and Solomon and Simret (2008). The total edible offals (2.8–3.2 kg) reported for HH (Asnakew and Berhan 2007) and Sidama goats (Tadesse et al. 2013) are also similar with finding of the present study. The fact that SS goats had lighter total offals in the present study indicates that this breed can produce more proportion of usable carcass components relative to their body size than Bati and HH goats.

Goats fed L2 had heavier liver than those fed L1, may be due to their higher nutrient intake and, consequently, greater deposition of reserve substances such as glycogen as noted by Mushi et al. (2009b). Because of their bigger body size, thicker skins and dense hair cover, HH and Bati goats produced heavier (p < 0.05) skin than SS goats. Similar observation has been noted by Kadim et al. (2003) on skins obtained from different Omani goat breeds.

## Conclusion

The study demonstrated the effect of genotypes and levels of concentrate on growth rate and slaughter characteristics of selected Ethiopian indigenous goats. Concentrate feeding at the rate of 1.5 % than 1 % BW provided adequate nutrients to support maintenance and growth rates of up to 51 g/day. It also resulted in heavier final body and carcass weights despite lack of influence on most of the body and carcass linear measurements. Among the three genotypes, HH goats had better yield and carcass weight with more developed muscles. The study further revealed that SS goats, though smaller in size, are capable of producing comparable carcass compared to Bati goats. This indicates the potential of SS goats to produce more carcass yield relative to their body size/weight. In general, the study indicates the potential of Ethiopian indigenous goat for meat production through supplementation of concentrate at the rate of 1.5 % body weight.

## Abbreviations

HH: Hararghe highland goat
SS: short-eared Somali goat
DM: dry matter
OM: organic matter
CP: crude protein
ADF: acid detergent fibre
NDF: neutral detergent fibre
BW: body weight
ADG: average daily gain
IW: initial weight
FW: final weight
FCR: feed conversion ratio
SBW: slaughter body weight
EBW: empty body weight
HCW: hot carcass weight
DP: dressing percentage
AOAC: Association of Official Analytical Chemists
GLM: general linear model
SAS: statistical analysis software
SEM: standard error of the means
GIT: gastro = intestinal tract
